# Synchronous motion predicts enhanced perceived human–robot teamwork

**DOI:** 10.1038/s41598-025-31050-w

**Published:** 2025-12-10

**Authors:** Filipa Correia, Pedro Marques-Quinteiro

**Affiliations:** 1 Interactive Technologies Institute, LARSYS, Lisbon, Portugal; 2 Instituto Superior Técnico, Universidade de Lisboa, Lisbon, Portugal; 3 Intrepid Lab, ECEO, Lusofona University, Lisbon, Portugal; 4 CETRAD, Centro de Estudos Transdisciplinares para o Desenvolvimento, UTAD, Vila Real, Portugal

**Keywords:** Human–robot teamwork, Human–robot synchrony, Synchronous behavior, Human behaviour, Electrical and electronic engineering

## Abstract

In social robotics, interaction synchrony plays a central role in creating intentional and lifelike robotic agents. However, is yet to be studied the extent to which interaction synchrony is a relevant social aspect used by external observers to make inferences about human–robot dyads. For instance, whether external observers evaluate the human–robot ability to work as a team. In one mixed-design experimental study, 34 participants were presented with two videos showing a human–robot dyad engaging in a synchronous vs. asynchronous interaction. We found evidence that the mere perception of synchronous interactions predicts external observers’ estimations of the dyad’s collective efficacy, fluency, and cohesion. Our findings also suggest that seeing asynchronous interactions after synchronous ones elicits greater differences in participants’ estimations, compared to when participants see asynchrony first. Unexpectedly, synchrony did not play a significant role in shaping participants’ affiliation intention towards the dyad. Overall, our findings speak to the importance of synchrony in shaping the way humans observe a human-robot dyad and think about their collaborative capabilities. Not only do we provide new insight into the way humans perceive social groups that include at least one robotic agent, but we also draw broader implications to the human–robot interaction field.

## Introduction

The interpersonal motor synchrony of human dyads is a phenomenon wherein individuals align their behaviors and movements during social interactions^1^. Previous studies have shown that this intricate coordination carries profound implications for those engaging in it^2^, and for those simply observing it^3^. In the first-person perspective, the reported effects range from feelings of affiliation^4,5^, increased attention towards partners^6,7^, to the performance of a collaborative task^8^. In the third-person perspective, the remarkable effects of synchrony observation include differences in the physiological state^9^, stronger perceived entitativity^2^, higher affiliation intentions^10^, and higher prosocial behaviors^11^. Hence, interpersonal synchrony plays a critical role in the development of human sociability, and our capacity to collaborate with others^1^.

In social robotics, interaction synchrony has long been reported as a central factor in creating intentional and lifelike robotic agents^12,13^. Previous research has focused on how can robots engage in coordinated actions with humans^14–16^, as well as how and when humans actually synchronize with robots^17–22^. For instance, people tend to prefer a robot with adaptive motor coordination capability compared to a robot without that capability^17^, and a robot with congruent gaze reactions is perceived with higher levels of likability and intelligence than a robot with incongruent reaction or no reaction^18^. However, little attention has been given to how synchrony is perceived in human–robot interactions specifically by external observers. This is particularly important as humans and robots often interact within broader social contexts where other humans may exist, who might in turn develop an expectation of teamwork based on the observed behaviors^1,18,23^.

Hence, this paper addresses this research gap by using an empirical methodology to evaluate human–human synchrony in the field of human–robot interaction, exploring the following research question: How do external observers rate a synchronous and an asynchronous human–robot dyad in terms of collective efficacy, affiliation intention, fluency, and cohesion? These variables are considered in the teamwork literature as primary enabling conditions for the unfolding of effective teamwork^24^, and we, therefore, anticipate that investigating the factors that enhance or hinder their development is as relevant to human–robot teaming.

## Results

To answer our research question, we conducted an online user study (N=34) in a mixed design with synchrony (i.e. synchronous or asynchronous behaviors) as a within-subjects factor, and the order (i.e. synchrony gain or synchrony loss) as a between-subjects factor. Participants were asked to watch two video stimuli of a human–robot dyad performing a synchronous and an asynchronous behavior and to rate their perceptions of each video. We measured participants’ subjective evaluations of the human–robot dyad in terms of collective efficacy (i.e. how individuals evaluate the overall team capacity to perform well on a collaborative task), affiliation intention (i.e. how much they would like to form a group with the dyad), fluency (i.e. the coordinated meshing of joint activities between members of a well-synchronized team), and cohesion (i.e. the extent to which the members of a group believe about the fact that they have a strong agreement about a shared goal).

### Between-subject effects

**Fig. 1:**
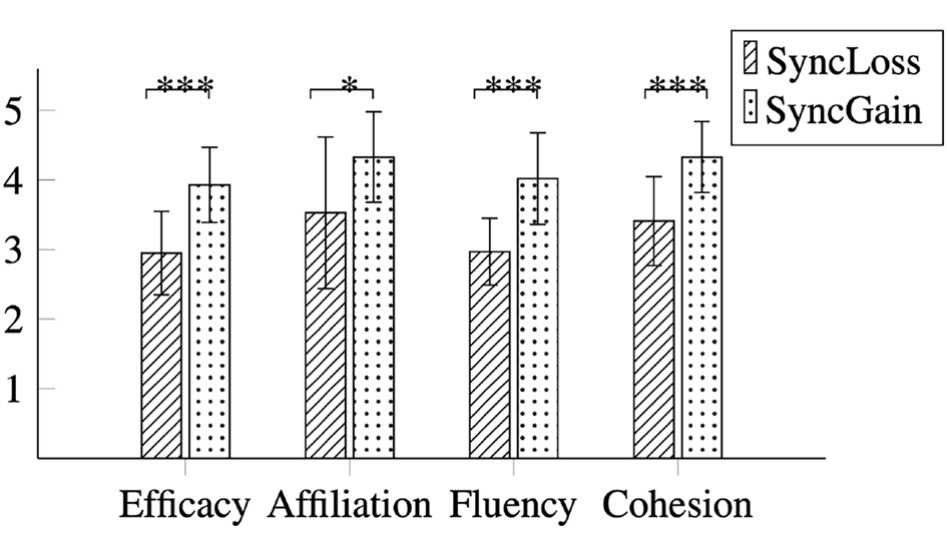
Mean values of the dependent variables collective efficacy, affiliative intention, fluency and cohesion attributed to both video stimuli in each ordering condition. Error bars are standard deviation values. ***$p<0.001$ *$p<0.05$.

We found significant differences between the order conditions on participants ratings, for collective efficacy, $F(1, 32)=24.75,p <.001, \eta _{p}^{2} =.44$, fluency, $F(1, 32)=28.00, p <.001, \eta _{p}^{2} =.47$, cohesion, $F(1, 32)=20.91, p <.001, \eta _{p}^{2} =.40$, and affiliation, $F(1, 32)=6.45,p =.016, \eta _{p}^{2} =.17$ (see Fig. 1). The average ratings of both videos were higher in the synchrony gain condition compared to the synchrony loss condition for all dependent measures. These results suggest that the capability of reaching synchrony between a human–robot dyad positively affects people’s estimations of the dyad’s attributes.

### Within-subjects effects

**Fig. 2:**
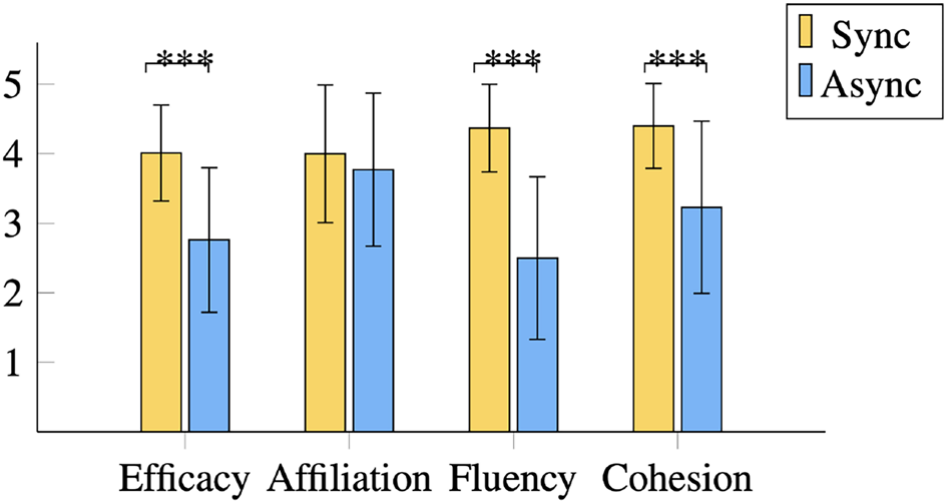
Mean values of the dependent variables collective efficacy, affiliative intention, fluency and cohesion attributed to the video stimuli displaying either human–robot synchronous behavior (sync) or asynchronous behavior (async). Error bars are standard deviation values. ***$p<0.001$.

We found a main effect of synchrony for collective efficacy, $F(1, 32)=68.73,p <.001, \eta _{p}^{2} =.68$, fluency, $F(1, 32)=132.97,p <.001, \eta _{p}^{2} =.81$, and cohesion, $F(1, 32)=27.65,p <.001, \eta _{p}^{2} =.46$. There was no main effect of synchrony on affiliation intentions, $F(1, 32)=3.91,p =.06, \eta _{p}^{2} =.11$ (see Fig. 2). The results showed that the mere observation of synchronous movements between a human and a robot leads people to attribute them a higher collective capacity to do well on a collaborative task, smoother coordinated actions, and higher union as a team, which is in line with previous literature on the observation of human–human synchrony^10^. However, we found no evidence that observing human–robot synchronous behavior affects individual’s willingness to affiliate with them, suggesting affiliative intentions towards human–robot teams might hold more intricate nuances when compared to human–human teams.

### Interaction effects

**Fig. 3:**
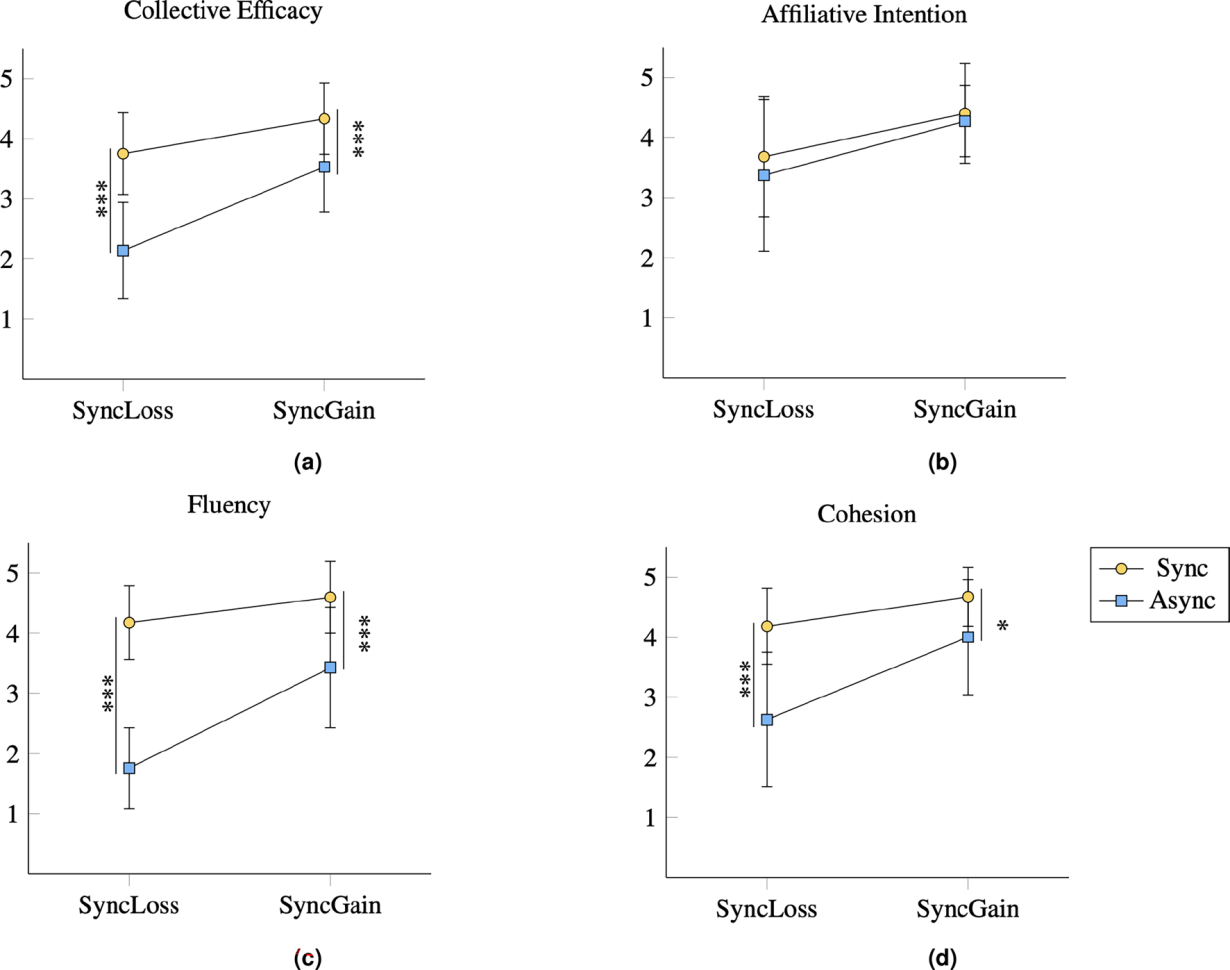
Mean values of the dependent variables collective efficacy (**a**), affiliative intention (**b**), fluency (**c**) and cohesion (**d**) attributed to the video stimuli displaying either human–robot synchronous behavior (sync) or asynchronous behavior (async), grouped by the ordering condition. Error bars are standard deviation values. ***$p<0.001$ *$p<0.05$.

The results of the within-between subject analysis also show a significant interaction effect between order and synchrony for collective efficacy, $F(1, 32)=7.71,p =.009, \eta _{p}^{2} =.19$, fluency, $F(1, 32)=16.26,p <.001, \eta _{p}^{2} =.34$, and cohesion, $F(1, 32)=4.41,p =.044, \eta _{p}^{2} =.12$ (see Fig. 3). There was no interaction effect between order and synchrony for affiliation intention, $F(1, 32)=0.65,p =.43, \eta _{p}^{2} =.02$. These findings suggest that the order by which participants saw a synchronous vs. asynchronous interaction had an effect in how they estimated the dyad’s attributes.

### Pairwise comparisons

Following-up on the interaction effects, we further examined the direction of the change in participants’ ratings within each condition. The outcome of the pairwise comparisons suggest that there was a significant mean difference between participants’ ratings, in such a way that there was a decrease in collective efficacy,* M*_*diff*_ = 1.07, SE = 0.19,* p* <.001, 95 CI [1.213, 1.998], fluency, * M*_*diff*_ = 2.42,SE = 0.21,* p* <.001 , 95 CI [2.000, 2.842], and cohesion, * M*_*diff*_ = 1.55, SE = 0.28, * p* <.001, 95 CI [0.982, 2.124], when participants saw synchrony first and asynchrony second.

Also, there was a significant increase in participants’ ratings of collective efficacy, * M*_*diff*_ = −0.80,SE = 0.22, * p* <.001, 95 CI [−1.242, −0.358], fluency, * M*_*diff*_= −1.17,SE = 0.23, * p* <.001, 95 CI [−1.640, −0.693], and cohesion, * M*_*diff*_ = −0.67,SE = 0.32, * p* =.042, 95 CI [−1.309, −0.024], when participants saw asynchrony first and synchrony second.

Overall, observers in the synchrony loss condition reported higher differences between the perceptions of synchronous and asynchronous human–robot movements in terms of collective efficacy, fluency, and cohesion, compared to observers in synchrony gain condition. Except for the dependent measure of affiliation, these results go in line with what happens in human–dyads^10^, showing synchronous movements become more salient whenever asynchrony follows synchrony.

### Additional analyses

Additionally, we decided to further explore our findings to add clarification to the process through which the perception of interpersonal synchrony in human–robot dyads can influence the perceptions and intentions of mere observers. Given the design of this study, we performed a repeated measures correlation analysis^25^, and repeated measures mediation analysis^26^.

The correlation between constructs was tested using the R package "rmcorr” for repeated measures correlations^25^. The results suggest that, with the exception of affiliation intention, all variables were positively correlated, $r(34)<.89, p <.001$.

The mediation analysis was performed using the SPSS macro MEMORE^27^. The results suggest that perceived interpersonal synchrony fully mediates the effect that synchrony vs. asynchrony has on observers estimations of collective efficacy, B = .99, SE = .26, 95 CI [0.556, 1.563], and cohesion, B = 1.22, SE = .33, 95 CI [0.662, 1.924]. Our findings also suggest that perceived interpersonal synchrony partially mediates the effect that synchrony vs. asynchrony has on observers estimations of fluency, B = 1.23, SE = .24, 95 CI [0.526, 1.182].

## Discussion

In the current study, we tested if the mere perception of synchronous vs. asynchronous interactions between one human and one robot triggers observers’ (i.e. external members) perceptions of collective efficacy, fluency, and cohesion of the human–robot dyad. Our results showed that observing synchronous movements between a human and a robot leads people to attribute them a higher collective capacity to do well on a collaborative task, smoother coordinated actions, and higher union as a team. Additionally, our findings suggest that the perception of synchrony plays an important role in how single individuals estimate the features and capabilities of human–robot dyads. These findings are in line with previous evidence from human–dyads research. If robots are integrating our society as collaborators and teammates alongside humans^28^, it becomes crucial to understand how observers perceive situations of human–robot teamwork. Our experimental results support that mere observation a synchronous movement between a human and a robot is able to affect the inferences people make about that human–robot dyad. Such results are, to our best knowledge, among the first systematic tests showing that just as synchrony plays an important role in modeling interpersonal relationships in humans and other social organisms, it is also relevant for modeling relationships between humans and robots. Generally, these reinforce the importance of synchrony as a social cue that drives the way humans think, feel, and act in a social context with robots.

Our results also suggest that human–robot synchrony is not straightforwardly perceived as human–human synchrony, it and holds particular nuances. For instance, most of our results go in line with previous literature on the impact of observing interpersonal synchrony^10^, except the willingness to affiliate with a human–robot dyad, in which we only found a significant difference of the between-subjects factor. Participants that saw the synchronous stimulus followed by the asynchronous stimulus reported higher willingness to affiliate with the human–robot team compared to the participants that saw the stimuli in the opposite order. This is particularly relevant for scenarios with autonomous robots that will engage with humans in continuous interactions that may hold both synchronous and asynchronous movements. When human–robot synchrony is reached, the possible following asynchronous movements will be negatively recognized, damaging the perception of teamwork by external observers. More broadly, it suggests the state of synchrony between humans and robots is expected to be sustained over time.

However, more investigation is needed to further understand how external observers develop affiliative intentions with teams of humans and robots. One possible explanation is that affiliating with a dyad that includes a robot may rely less on momentary interpersonal cues, such as synchrony, and more on broader group-level categorizations. From a social identity perspective, observers might not spontaneously categorize human–robot teams as a unified ingroup^29^, which may limit the extent to which synchrony can boost interpersonal affiliation. This possibility aligns with theories of group identity and self-categorization, which predict that affiliative intentions depend on whether observers perceive a shared social identity with the agents involved^30^. Another complementary interpretation is that order effects may reflect inferences about the robot’s underlying characteristics, such as its competence^31^. Observers often use behavioral cues to form judgments about an agent’s internal capacities. When participants first observe asynchrony, they might infer that the robot is less competent or less capable of coordinating with a human partner; this negative impression can anchor subsequent evaluations.

Furthermore, we observed that the order affects the perception of the two synchronous stimuli. Observers in the synchrony loss condition reported higher differences between the perceptions of synchronous and asynchronous human–robot movements in terms of collective efficacy, fluency, and cohesion, compared to observers in synchrony gain condition. Similarly to human–dyads, synchronous movements become more salient whenever asynchrony follows synchrony.

Lastly, we found significant between-subjects effects of the order on all dependent measures. The average ratings of both videos was higher in the synchrony gain condition compared to the synchrony loss condition. On the one hand, the salience of synchrony following asynchrony is consistent with well-documented contrast effects^32^, as previously experiencing low coordination can make subsequent synchrony appear especially positive, thereby elevating evaluations. On the other hand, expectation-based mechanisms might also support this result^33^, considering that once synchrony is established, participants may develop an expectation that coordinated behavior will be maintained, while violations of this expectation, such as a shift to asynchrony, can be judged more harshly due to expectancy violation processes. This finding is particularly relevant for autonomous robots that will engage with humans in continuous interactions that may hold both synchronous and asynchronous movements. When human–robot synchrony is reached, the possible following asynchronous movements will be negatively recognized, damaging the perception of teamwork by external observers. More broadly, it suggests the state of synchrony between humans and robots is expected to be sustained over time.

## Broader implications

Research on human collaboration and teamwork has systematically highlighted the importance of developing collective efficacy beliefs and cohesion, to enable healthy and productive relations. Humans often enjoy working in teams or being in groups that are perceived as being more competent at what they do, and which see themselves as a cohesive unit^24^. Our research findings show that these properties can be inferred by others who are not part of the team or group, which is consistent with prior research^3^. It further reinforces the importance of synchrony as a social cue that drives the way humans think, feel, and act in a social context with robots.

Following on the previous implication highlighting the importance of further investigating human–robot synchrony, we would also like to draw attention to our new video stimuli of synchronous and asynchronous human–robot behaviors. We validate the two developed video stimuli regarding the perceived synchrony between the human–robot dyad. These materials contribute to the research community as reusable instruments to further analyze phenomena associated with human–robot synchrony by external observers.

Additionally, if robots are integrating our society as collaborators and teammates alongside humans^28^, it becomes crucial to understand how observers perceive situations of human–robot teamwork. As we become surrounded by social robots in several domains (e.g. homes, hospitals, schools, workplaces), we establish and create powerful associations simply observing those robots. Our experimental study provided support that mere observation a synchronous movement between a human and a robot is able to affect the inferences people make about that human–robot dyad.

Our findings also have implications for the design of social robots, or robots whose purpose involves some form of interaction with humans. If designed to be immersed within a social context, these robots will be seen and appraised by humans. Designing them in ways that enable the synchronization of bodily movements, may increase the robots’ ability to convey social cues that are positively perceived by humans and therefore increase robots’ acceptance and integration.

### Limitations and future directions

Like any research, the current study is not without limitations. Hence, there are three important considerations in our experimental design to take into account when generalizing our findings. First, the chosen robot can be considered highly anthropomorphic as it possesses a torso, head, and face. As robotic embodiments have an influence on human attributions of likability and trustworthiness regarding the robot^34^, a future extension of the current study could be the testing of robot anthropomorphism as a boundary condition to the observed effects of interpersonal synchrony on the perception of human–robot and robot-robot dyads^35^. Likewise, task-irrelevant social information such as the skin color, the dressing, or the gender of the dyad’s human actor, should also be considered as these elements have been previously found to bias observers’ estimations regarding interpersonal synchrony^3^.

Second, although the human–human dyad literature has provided consistent evidence regarding the effects of interpersonal synchrony on both dyad members and bystanders’ perceptions of dyads’ attributes, the present study would have benefited from the inclusion of two additional control conditions: a human–human dyad and a robot–robot dyad. A human–human condition would have allowed for the replication of previous findings, serving as an additional validity check for the experimental manipulation and ensuring that the stimulus materials effectively elicited interpersonal synchrony as intended. In turn, a robot–robot condition could have tested the consistency of the findings in the absence of human agents and opened avenues to further explore the role of anthropomorphism in shaping bystanders’ perceptions of dyads engaged in synchronous versus asynchronous interactions.

Third, our choice of synchronous behavior was the head movements because this is the simplest to generalize across a broader variety of robotic embodiments^36^, and because nodding has been systematically and consistently identified as a basic synchronization process through which external observers obtain implicit cues about the level of synchrony between two interacting agents^37^. However, there are an abundance of bodily movements that can be explored regarding interaction synchrony, including finger tapping, and arm, leg, and body sways, for all of which the manipulation of synchronous and asynchronous interactions is achieved by converging and diverging the rhythm of dyads’ members movement^1^. To the best of our knowledge, there is no scientific evidence indicating that the synchronization of different bodily parts elicits distinct perceptions of the same social cues, or that such synchrony is uniquely associated with specific social cues. Nevertheless, it remains important to investigate whether different robotic embodiments might enable the exploration of other forms of synchrony–or even extend beyond movement to include behaviors such as vocal patterns or emotional expressions–and to assess the stability of the phenomenon.

Finally, building on previous research by Cirelli et al., the findings from our study could also be used to inform future studies on larger groups with three or more team members performing an interdependent task with a shared goal; and human child-robot interactions. In Cirelli et al., the authors found that toddlers involved in synchronous bouncing with perfect strangers display more prosocial (as helping) behaviors than those involved in asynchronous bouncing^38^. The prosocial aspect in this study involved a brief moment of affiliation, where the toddler spontaneously decided to help the experimenter. It would be interesting to explore a landscape where toddlers were mere observers of human–robot dyads’ synchronous vs. asynchronous interactions, and how their perceptions would shape subsequent behaviors.

## Methods

### Stimuli development and validation

As there were no prior stimuli materials that we could use for the current research, the first step was to develop them. Our goal was to create two short video stimuli of a human–robot dyad either performing synchronous or asynchronous behaviors in a similar context and situation. The chosen coordinated actions to prompt either synchrony or asynchrony between the human–robot dyad were head movements because (a) these are the simplest to generalize across a greater variety of robotic agents (considering the majority of robots used by the scientific community in Human–Robot Interaction^36^), and (b) head movement, i.e. nodding, has been systematically identified as a core feature of interpersonal movement synchrony, allowing external observers to perceive and interpret implicit cues about the degree of coordination between two interacting agents^37^.

**Fig. 4:**
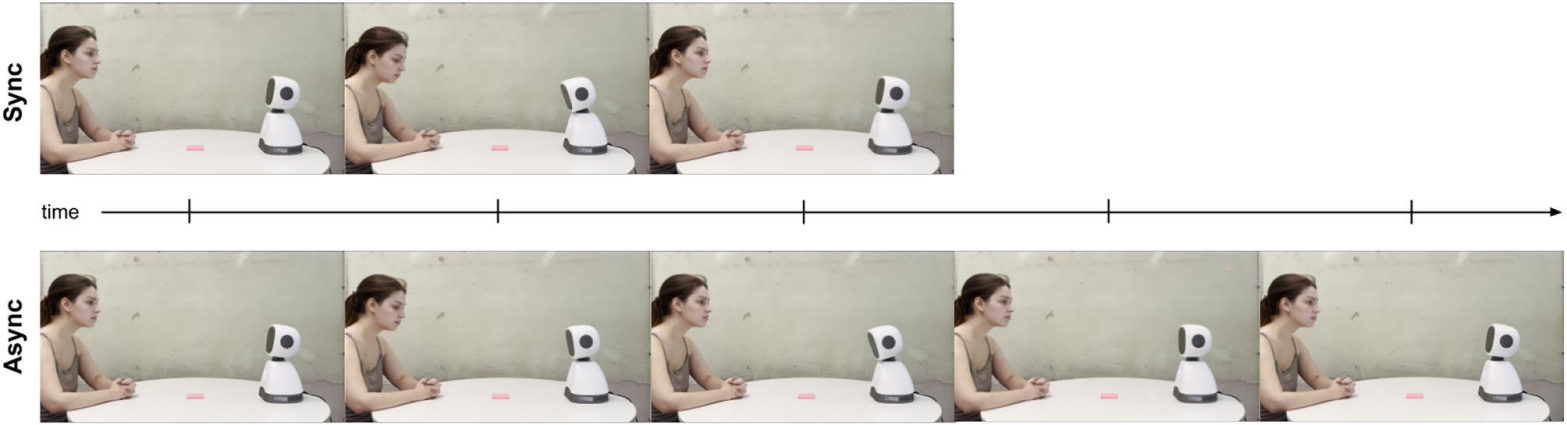
Frames of the video stimuli for each condition.

The video stimuli portray a human and an Elmo robot on a round table gazing at each other. In both videos, they perform two head movements: gazing at the object on the table and then gazing back at each other. The object on the table is a practical representation of the shared goal between the human and the robot, an idea that is reinforced in the information that is passed to the research participants when they are taking the study. The difference between the two videos is their velocity to perform these head movements. In the synchronous stimulus, the human and the robot have the same velocity performing both movements in $\approx$2 seconds, and they start and finish the movements at the same time. While in the asynchronous stimulus, the robot performs the movements in $\approx$4 seconds and, therefore, they start at the same time but the robot finishes later than the human (see Fig. 4). The human in the videos voluntarily accepted to act accordingly for each recording and gave their informed consent beforehand.

The decision to use velocity as a variable to manipulate interpersonal synchrony is closely related to the operationalization of interpersonal synchrony as the temporal coupling of bodily movements between two or more agents^1^. While synchronous interactions entail the perception that individuals’ movements happen with a similar rhythm (as speed), asynchronous interactions entail the perception that individuals’ movements happen in different rhythms.

The movements of the robot and the human were recorded separately and merged using the software DaVinci Resolve 18.1.4 with a keyframe mask that overlaps videos only in a predefined portion of the window. The movements of the human were following a metronome with a beat of 60 BPMs for both video stimuli. The movements of the robot were scripted and correspond to the beats of 60 BPMs for the synchronous video, and 120 BPMs for the asynchronous video. This artificial development of the stimuli was crucial for the asynchronous video because when we initially tried to record both behaviors on the same scene, the human had a tendency to follow the robot instead of the metronome. For the synchronous stimulus, the artificial manipulation of videos allowed us to do adjustments of milliseconds ensuring the exact same duration of each movement for both the human and the robot.

Once the materials were made, we performed a validation of both stimuli in terms of the perceived level of synchrony. Participants were recruited through social media to answer an online survey, in which they saw ad rate both video stimuli in a within-subjects design. We computed the necessary sample size using G*Power 3.1.9.2^39^ for a power analysis on a paired sample design (t-test, differences between two dependent means, matched pairs). The input parameters were the default: effect size *dz* = 1.00, $\alpha$ = 0.05, power = 0.95. This analysis suggested that, for a critical *t* of 2.13, at 95 per cent power, a sample size of 16 was required (actual power = 0.96). Therefore, we recruited 16 volunteers to answer the online survey, *M*_age_ = 33.31, *SD* = 10.11, of which 8 were women and 6 had at least a degree. Ten participants were randomly assigned to watch the synchrony stimuli first, and six participants were randomly assigned to observe the asynchrony stimuli first.

To assess the perceived level of synchrony between the human–robot dyad, we used 3 items, adapted from^40^. The items were "There was synchrony in the way the human and the robot interacted”, "The human and the robot moved in the same way”, and "The rhythm with which the human and the robot moved was the same”. The 3 items had good reliability, $\alpha$ = .89, and were, therefore, aggregated into a single measure of synchrony. Participants did not receive any instruction prior to visualizing the videos, except that they were requested to pay attention to the videos and that each video would play only once.

The mean score for the synchrony video was *M*=4.79, *SD*=.42, while the mean score for the asynchrony video was *M*=2.29, *SD*=.74. The results of the paired sample t-test analysis where statistically significant, *t*(15)=12.53, $p <.001$, which shows that participants were able to differentiate between the two videos based on the dyad’s synchrony. This results supports the adequacy of the two videos as stimuli material for the current study.

### Procedure and research design

In line with the primary goal of this research, we followed the same study design of Marques-Quinteiro et al.^10^. Differently, our design focused on human–robot interaction without including a parallel human–human control condition. This choice was informed by the research findings in Marques-Quinteiro et al. which display robust effects of interpersonal synchrony in human–human settings. The authors conducted four experimental studies, complemented by an internal meta-analysis, in which participants observed synchronous and asynchronous dyads. Across these studies, the effects of synchrony on observers’ perceptions were replicated consistently, underscoring the reliability of the phenomenon in human–human contexts. Building on this well-established evidence base, the present study extends the investigation of synchrony into the domain of human–robot interaction, using the human–human findings as a conceptual benchmark.

Participants were recruited to do an online survey to watch and rate two videos of a collaborative task between a human and a robot. The study had a mixed design with synchrony as the within-subject factor and the order as the between-subject factor. Synchrony had two levels corresponding to the two video stimuli of a synchronous and an asynchronous human–robot dyads. For the between-subject factor, participants either saw synchronous stimulus followed by the asynchronous stimulus (i.e. synchrony loss condition), or the asynchronous stimulus first followed by the synchronous stimulus (i.e. synchrony gain condition). Participants were randomly assigned to one order condition (i.e. synchrony loss or synchrony gain), which determined which video stimuli was seen first.

Participants saw each video only once, which was programmed to play automatically. Before each video, participants were told to "Think about the video you just watched. Imagine that the human and the robot were working together on a collective task”, and then provide their own estimates on the dyad’s **collective efficacy** (4 items, Chronbach $\alpha$ synchrony = .70, Chronbach $\alpha$ asynchrony = .91; one item example is "the human and the robot had good performance”)^41^; **fluency** (2 items, Chronbach $\alpha$ synchrony = .58, Chronbach $\alpha$ asynchrony = .80; one item example is "the human and the robot worked fluently together”)^42^; **cohesion** (2 items, Chronbach $\alpha$= synchrony = .67, Chronbach $\alpha$ = asynchrony = .72; one item example is "the human and the robot had one shared goal”)^43^; **affiliation intention** (1 item, "Given the opportunity, I would help the human and the robot doing their task)^10^; and perceived synchrony (3 items, Chronbach $\alpha$= synchrony = .88, Chronbach $\alpha$ asynchrony = .92; one item example is "There was synchrony in the way the human and the robot interacted”)^39^. The responses to all items used a Likert type-scale ranging between 1 (very unlikely) and 5 (very likely). Participants received a reward of 1.5 GBP, following the recommended hourly rate of 9 GBP and the initial estimate that it would take 10 minutes. The median time to complete the survey was however 4 minutes and 14 seconds.

It is important to note that the collaborative activity of the target dyad was deliberately framed in the most general terms possible to minimize contextual factors (e.g., industry type, economic activity) and task-specific characteristics (e.g., task nature, task goals) that might confound the findings. This approach also helped reduce the risk of unnecessary demand effects, thereby safeguarding our primary objective of examining interpersonal synchrony as an independent variable^44,45^.

The experimental procedure was approved by Ethical Committee of the Lusofona University (Ref: CE ILIND Ata 12), and the experiment was performed in accordance with relevant guidelines and regulations.

### Robot selection and specifications

We chose the Elmo robot, a prototype developed at IDMind company, as the robotic device to serve as an embodied artificial agent in the human–robot interaction. This robot has 2 degrees of freedom at the neck joint, allowing it to move its head with pan and tilt movements. Therefore, this tabletop robot has some anthropomorphic features. The head has a display usually showing cartoonish eyes; however, in our experiment, because of the chosen side position, the eyes could not be perceived by observers of the videos. Regarding the level of anthropomorphism, which was not assessed in our experiment, the Human–Likeness Predictor of the ABOT Project^36^ estimates a score of 24.97 considering the characteristics of this robot. This score is alligned to the robots Buddy, Zenbo or Yumi True Smart, which have similar features to Elmo.

### Participants

Participants were 34 individuals, recruited using the Prolific platform on July 12th, 2023, and they received informed consent about their participation. Participants had *M*_age_ = 28.38, *SD* = 8.40, of which 16 were women and 13 had at least a degree. We computed an a priori power analysis using G*Power 3.1.9.2^39^ for a mixed design (F-test, repeated measures, within-between interaction), and selected the procedure based on effect from direct variance, considering the partial eta squared ($\eta _p^2$ = .05) calculated from the information available in^10^. The input parameters were: effect size f = 0.25, $\alpha$ = .05, power = .80, groups = 2, measurements = 2, correlation among repeated measures = .25, non-sphericity correction = 1 (default). This analysis suggests that, for a critical *F* of 4.15, at eighty percent power, a sample size of 34 was required (actual power = 0.82). Nineteen participants were randomly assigned to the synchrony loss condition, and fifteen participants to the synchrony gain condition.

### Manipulation check

The perception of synchrony was measured as a manipulation check, in order to determine if participants could differentiate between the two regarding the synchronous and asynchronous stimuli of the dyad’s interaction. Our findings suggest a significant difference between participants ratings in the order condition, *F*(1, 32) = 50.81,* p* <.001, $\eta _{p}^{2}$ =.61, as well as a main effect of synchrony,* F*(1, 32)=162.12,* p* <.001, $\eta _{p}^{2}$ =.84, in participants estimations of dyad interpersonal synchrony. Additionally, there was a significant interaction effect between order and synchrony,* F*(1, 32) = 38.37, * p* <.001,$\eta _{p}^{2}$ =.55.

The outcome of the pairwise comparisons also suggests that interpersonal synchrony perceptions decrease when participants see synchrony first and asynchrony second, * M*_*diff*_ = 3.28, SE = 0.23, *p* <.001, 95 CI [2.812, 3.750], and decrease when participants see asynchrony first and synchrony second, * M*_*diff*_= −1.33, SE = 0.26, *p* <.001 , 95 CI [−1.661, −0.605].

Together, these results suggest that the videos worked as stimuli material to experimentally manipulate observers’ perceptions of interpersonal synchrony as intended.

## Data Availability

The datasets generated during and/or analysed during the current study are available in the OSF repository, https://osf.io/znu3a/.
